# Characterization of Buoyant Fluorescent Particles for Field Observations of Water Flows

**DOI:** 10.3390/s101211512

**Published:** 2010-12-15

**Authors:** Flavia Tauro, Matteo Aureli, Maurizio Porfiri, Salvatore Grimaldi

**Affiliations:** 1 Department of Mechanical & Aerospace Engineering, Polytechnic Institute of New York University, Brooklyn, NY 11201, USA; 2 Honors Center of Italian Universities (H2CU), Sapienza University of Rome, Rome 00184, Italy; 3 GEMINI Department, Agricultural School, University of Tuscia, Viterbo 01100, Italy

**Keywords:** fluorescent particles, hillslope processes, hydrologic tracers, processing of sensed data, sensors’ applications

## Abstract

In this paper, the feasibility of off-the-shelf buoyant fluorescent microspheres as particle tracers in turbid water flows is investigated. Microspheres’ fluorescence intensity is experimentally measured and detected in placid aqueous suspensions of increasing concentrations of clay to simulate typical conditions occurring in natural drainage networks. Experiments are conducted in a broad range of clay concentrations and particle immersion depths by using photoconductive cells and image-based sensing technologies. Results obtained with both methodologies exhibit comparable trends and show that the considered particles are fairly detectable in critically turbid water flows. Further information on performance and integration of the studied microspheres in low-cost measurement instrumentation for field observations is obtained through experiments conducted in a custom built miniature water channel. This experimental characterization provides a first assessment of the feasibility of commercially available buoyant fluorescent beads in the analysis of high turbidity surface water flows. The proposed technology may serve as a minimally invasive sensing system for hazardous events, such as pollutant diffusion in natural streams and flash flooding due to extreme rainfall.

## 1. Introduction

Watershed surface processes control downstream runoff phenomena [1,2], waste and pollutant diffusion [3], erosion mechanics [4,5], and sediment transport [6,7]. These flows are largely dominated by ephemeral microchannel drainage networks in hillslope areas [8–11]. A quantitative understanding of the flow physics in these areas is currently limited by the lack of effective tracing techniques suitable for basin-scale observations [12]. More specifically, field experiments require environmentally resilient, non-invasive, and low-cost measurement systems that can potentially operate in remotely-controlled or unmanned conditions. Furthermore, water turbidity, flow path heterogeneity, and natural flow obstructions impose severe constraints on sensing technologies in field studies [13,14].

Traditional tracing methodologies are largely not capable to cope with extreme in-situ conditions, including practical logistic challenges as well as inherent flow complexity [15,16]. Most of available technologies need physical sampling to estimate the tracer concentration and do not allow for continuous-time measurements [17–19], which are crucial in understanding the evolution of hydrologic phenomena. In addition, commonly used tracers, such as isotopes, dyes, and chemicals, are not directly applicable to monitor surface hillslope processes and large-scale microchannel networks due to elaborate detection processes and dispersion issues [12,15,18,20–25]. Most of these traditional tracing methodologies tend to infer global parameters from local measurements [15] and are not generally capable of capturing the fast evolution of processes at the watershed-scale [26]. On the other hand, feasibility studies on emerging technologies in the study of overland flows, such as tracing particles and drifting buoys, are presently not available. Moreover, the bulkiness of such devices restricts them to channel flow tracking and oceanography applications [27,28].

Related research on Particle Image Velocimetry (PIV) in fluid dynamics [29] and hydrology [30] has also fueled the design and characterization of novel particle tracers for flow studies [31]. Among this class of beads, fluorescent particles show high efficiency and detectability at almost every flow velocity and water depth [32,33]. The synthesis of dedicated fluorescent particles for PIV laboratory-scale studies is reported in [32]. Fluorescent polymer nano- and microspheres have been used to study near-wall fluid motion [34] and mixing processes in multiconstituent and multiphase fluid systems [32]. In biomedicine, vision-based methodologies rely on the enhanced visibility of fluorescent corpuscles in turbid media to identify and isolate proteins and pathogens in living organisms [35–38]. In addition, controlled fluorescence microspheres are used in Magnetic Resonance Imaging (MRI) for tumor detection and medical imaging [39].

Despite its promise, the use of fluorescent microparticles in hydrologic research is largely limited to flow studies in small-scale laboratory experiments. The only example of fluorescent particles designed for potential in-situ Large Scale PIV (LSPIV) measurements is reported in [40]. It is therein shown that reduction of reflections due to the presence of free surfaces, sediments, and interfaces is a remarkable advantage of fluorescent particles in large-scale hydraulic experiments. In particular, in [40], a novel particle tracer is synthesized by mixing Rhodamine Water Tracer (WT), a low toxicity fluorescent dye, with commercial grade liquid polyester resin with a density of 1.2 g/cm^3^. Upon curing, the resin is formed into a block and subsequently ground into a powder. The powder is then sieved to extract particles smaller than 63 μm for experiments. The tracer is detected through common PIV and, due to the properties of the fluorescent material, good images are obtained in the vicinity of laser-reflective surfaces. However, use of these particles in underwater operations may be limited by the porosity of the resin material. Indeed, after their release, particles may absorb water and rapidly increase their density, as reported in [41] for polymer-based composites. Therefore, in the long run, beads may lose their natural buoyancy and precipitate.

In this paper, the feasibility of off-the-shelf buoyant fluorescent microspheres as particle tracers in turbid water flows is investigated. Microspheres’ fluorescence intensity is experimentally measured and detected in placid aqueous suspensions of increasing concentrations of clay to simulate typical conditions occurring in natural hillslope drainage micronetworks. More specifically, experiments are conducted for different levels of clay concentration ranging from 0 g/L to 60 g/L. The largest concentration corresponds to a remarkably high level of mountain-stream suspended sediment load, occurring during heavy floods [42]. Clay is selected for its fine size (10^−6^ to 10^−11^ m, see [43]) that results in high turbidity and slow sedimentation. Moreover, the particle visibility is studied at various immersion depths to account for the effect of turbulent flows which may tend to drag particles under the water surface, thus limiting their detectability [44,45]. Measurements are performed by following two different schemes that provide a thorough understanding of the potential and limitations of commercial particle tracers in field observations. The former measurement method is based on direct fluorescence intensity measurement through an array of photoresistors; the latter scheme entails image-based detection of the considered beads. Additional information on particle performance and integration in low-cost measurement instruments for field observations is garnered through experiments conducted in an in-house developed miniature water channel. This experimental characterization aims at providing an assessment of off-the-shelf fluorescent beads performance in tracing high turbidity surface water flows.

The proposed fluorescent particle tracer represents a low-cost, versatile, and non-invasive detecting technology for high sediment load conditions. Differently from traditional practice, this methodology is suitable for continuous and fully automated measurements that do not require sampling and human supervision. In addition, dispersion issues are expected to be negligible, thus promoting beads’ deployment in watershed-scale studies. In view of these advantages, the particle tracer is expected to both enhance the surface water flow observation practice and contribute to the advancement of cutting edge hydrologic research, such as runoff modeling, hydrochory diffusion [46,47], and river restorations [48].

The rest of the paper is organized as follows. In Section 2, materials and methods used in this study, including the experimental set-ups used for direct intensity measurements and image-based analysis in clay suspensions, are presented. In Section 3, results from the experimental campaign are discussed and compared. In Section 4, the potential of the proposed tracer technology for in-situ applications is discussed and technological aids for transitioning into field studies are presented. Section 5 is left for conclusions and future research directions.

## 2. Experimental Methods

The considered tracing methodology entails the detection of fluorescent particles in turbid water flows. The fluorescence response of the buoyant beads in realistic conditions is simulated by dispersing them in suspensions of water and clay. Concentration of clay is varied in a broad range to replicate different levels of turbidity. Fluorescence response is characterized by alternatively using a photoresistors’ array and analyzing images collected from a CMOS camera. In both experiments, the water is kept motionless and the photoresistors’ array and the camera are located below the suspension of particles and clay. This arrangement aims at simulating in-situ conditions, where buoyant particles may reside below the turbid water surface due to the flow regime. Before each experimental acquisition, care is taken in avoiding undesired clay sedimentation by periodically stirring the suspension.

An 8 W Ultra Violet (UV) 365 nm wavelength lamp located above the particles excites the fluorescence at a distance kept constant within each experimental scheme. Throughout the full set of experimental methodologies, this distance is adjusted in the range of 5–40 cm to account for the inherently different characteristics of the distinct sensing technologies and experimental scenarios. Experiments are conducted by varying both the clay concentration in the suspensions and the particle immersion depth from the water surface.

In field studies, both excitation light and camera are expected to be placed on the same side with respect to the water surface. Therefore, in natural environments, particle fluorescence intensity varies not only due to the bead immersion depth and turbidity but also with the distance from the light source. However, in this paper, particle fluorescence is not regarded as a parameter and is kept constant throughout the experiments by illuminating the beads at a fixed distance. In particular, the decrease in particle fluorescence intensity due to water scattering of the UV source is not considered. Therefore, particle detectability is only studied as a function of the microsphere’s immersion depth and turbidity.

### 2.1. Materials

**Figure 1.**
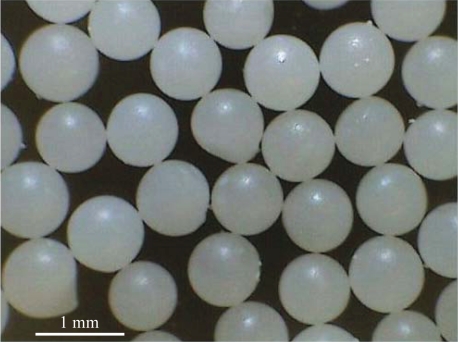
Particle microscopy showing size dispersion and system geometry.

The particles are purchased from Cospheric LLC [49] and their cost is approximately $100 for a 10 g sample; cost is reduced to $500/kg for large batches. The beads are approximately spherical and their diameter is in the range of 0.710–0.850 mm, as displayed in the microscopy in Figure 1. The spheres are white under daylight and emit yellow-green light (561 nm wavelength) if excited by a UV light source (365 nm wavelength). The particle material is polyethylene and the fluorophore is embedded in the polymer matrix which allows for a long luminescence life-time. The particles nominal dry density is 0.99 g/cm^3^.

### 2.2. Measurement of Particle Fluorescence

**Figure 2.**
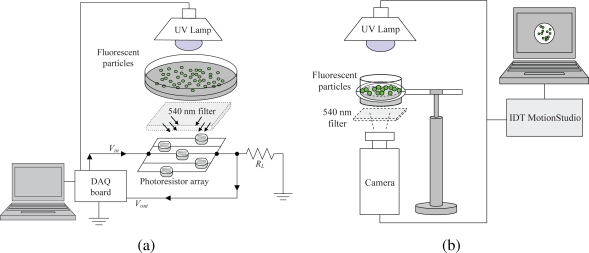
Schematic of the measurement system for (a) characterization of particle fluorescence and (b) particle detection.

Particle fluorescence in a turbid water flow is characterized by using the experimental set-up in Figure 2(a). A set of 100 fluorescent particles are deployed in a plastic 5 cm diameter Petri Dish, thus covering a surface area fraction of the container of approximately 2.55%. The system consists of a voltage divider circuit placed 5 cm below the particle container and interfaced with a computer through a National Instruments NI USB-6221 Data Acquisition (DAQ) Board and an in-house developed Labview Virtual Instrument (VI). The circuit comprises an array of five PerkinElmer (St. Louis, MO, USA) VT900 photoconductive cells in parallel connection and one load resistor *R_L_* in series to the photocells. A 540 nm filter is placed between the particle container and the photoresistors to isolate bead emissions from UV light. The slight mismatch between the particle emission and the filter wavelengths, 561 nm against 540 nm, does not compromise the fluorescence intensity measurement. In addition, the experimental set-up is enclosed in a dark environment to avoid noise effects due to external light. The nominal resistance of each photoresistor under UV emission is on the order of 1 MΩ. Thus, a load resistance *R_L_* = 200 kΩ is selected to match the array resistance and improve on the measurement sensitivity as per the voltage divider configuration [50].

A Direct Current (DC) voltage of 5 V is applied to the series resistors to detect the change in resistance of the photoresistors under varying light intensity conditions. The voltage across the load resistor *R_L_* is acquired at a sampling rate of 100 Hz and the resistance change is indirectly determined using [50]

(1) $$V_{\text{out}}=\frac{R_{L}}{R_{\text{Pr}}+R_{L}}V_{\text{in}}$$

where *V_out_* and *V_in_* are the voltages measured and supplied through the DAQ board, respectively, and *R_Pr_* denotes the resistance of the parallel connection of the photoresistors’ array.

The use of a photoresistors’ array against a single photoresistor is motivated by the need for reducing measurement noise and minimizing the effects of dishomogeneity in the particle dispersion within the Petri Dish. Furthermore, a fair repeatability of the experiments is guaranteed by maintaining the UV light at a constant distance of 5 cm from the sample and by placing an opaque separator between the lamp and the particle container. This allows the photoresistors to capture only the light that filters through the sample. Fluorescence characterization is performed in this test by selecting a constant level of the suspension in the container, that is, a constant particle immersion depth.

### 2.3. Image-Based Detection of Particles

Results from the photoresistors’ array can be validated by and complemented with data from image analysis techniques. In particular, information on particle location in the environment can be garnered by using image-based analysis tools. An IDT MotionPro 3 Series 1 k × 1 k pixel color CMOS camera fitted with a 540 nm optical filter for image acquisition is placed below the particle container as displayed in Figure 2(b). The camera is placed at the constant distance of 30 cm below the particle container. Beads are illuminated by the UV lamp located 10 cm above the particles. An opaque separator is also inserted between the lamp and the container to avoid light that does not filter through the sample to be captured by the camera. In addition, the camera experimental set-up is also enclosed in a dark environment to avoid noise due to surrounding light sources. Images of increasing concentrations of clay-water suspensions are collected by the camera at a sampling frequency of 5 Hz and with an exposure time of approximately 0.2 s. These parameters, along with image settings as brightness, contrast, and gamma correction, are kept constant during the experiments to allow for a proper comparison among different concentrations. The ratio of the bead pixel area to the analyzed frame area in the camera experimental set-up is kept equal to 2.55% to allow for a fair comparison with findings from particle fluorescence measurements.

In addition to the information provided by the use of photoresistors, the camera-based system allows for visual bead identification and localization in the area of interest. Particle size and shape can be recognized by using traditional image processing tools, such as edge detection [51]. Furthermore, information on the presence of fluorescent features can be obtained by analyzing the digital image histogram, that is, the discrete function *h*(*c_k_*) = *n_k_* where *c_k_* is the *k*-th intensity level and *n_k_* is the number of pixels in the image whose intensity level is *c_k_*, see [51].

## 3. Results

The experimental campaign focuses on the particle visibility in turbid aqueous suspensions and at different bead immersion depths below the water surface. More specifically, for a fixed immersion depth, fluorescence visibility is tested in suspensions of increasing clay concentrations from 0 g/L to 60 g/L by using the photoresistors’ array. In addition, influence of immersion depth on particle detectability is investigated by using the camera-based experimental set-up and then processing the recorded frames. In this case, immersion depths are varied from 4 to 10 mm with increments of 2 mm.

### 3.1. Measurement of Particle Fluorescence

**Figure 3.**
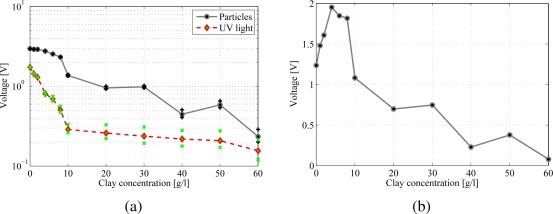
(a) Particle fluorescence and UV light intensity for increasing clay concentrations and (b) corresponding difference between photoresistors’ response to particle and UV light intensity.

Particle fluorescence is measured by using the photoresistors’ array. Experiments are conducted by varying the clay concentration while holding constant the number of particles to 100 and the immersion depth to 4 mm. For each experiment at a given concentration, two or three independent measurements consisting of voltage data sampled at 100 Hz in nine seconds are collected and analyzed to provide statistical significance. More specifically, for each set of 900 samples, a mean value is computed and further averaging of these mean values yields the photoresistors’ array output voltage displayed in Figure 3(a). In Figure 3(a), the black solid line represents the photoresistors’ output voltage when the UV light illuminates both the suspension and the particles. The dashed red line shows the output voltage when the UV light illuminates only the clay suspension in the absence of particles. Black and green markers in the plot identify maximum and minimum average values from each experiment. As the clay concentration increases, less light is transmitted through the suspension and the recorded output voltage decreases. Voltage values corresponding to the UV light intensity, shown as a dashed red line, decrease with clay concentration, exhibiting a trend similar to the trend relative to the particle intensity, displayed as a solid black line. In Figure 3(a), the dashed red line is always below the curve corresponding to particle fluorescence, demonstrating that the beads can be sensed at every clay concentration. For further analysis, the difference between particle and light intensity is plotted in Figure 3(b). The peak of the curve corresponds to a 4 g/L clay suspension.

Two additional experiments are performed to estimate the influence of reflections and scattering from the container on the overall visibility of the beads. In the first experiment, the photoresistors’ response to UV light in presence of the empty Petri Dish, is measured. In the second experiment, the photoresistors’ response to particle fluorescence is measured by deploying 100 beads in the empty dish. The difference between the two output voltage signals is approximately equal to 0.86 V, not shown in Figure 3(b). This value is lower than the value 1.2 V in Figure 3(b) corresponding to 0 g/L, indicating that the presence of water magnifies the particle visibility.

### 3.2. Image-Based Detection of Particles

Particle detection is performed by using the image-based experimental set-up. In this arrangement, a single particle is introduced in the Petri Dish for vision-based analysis. Particle detection is performed while varying both the clay concentration and the bead immersion depth. In clear water conditions, geometrical and optical characteristics of the system yield a typical bead reference frontal area of 9 × 9 pixels. The size of the analyzed image is then set at 56 × 56 pixels to keep a constant ratio of particle area to frame area approximately equal to 2.55%. A total of ten frames is recorded for each concentration and immersion depth and one randomly selected image is processed. Additionally, so-called background frames are collected by acquiring pictures of the clay suspension without fluorescent particles.

**Figure 4.**
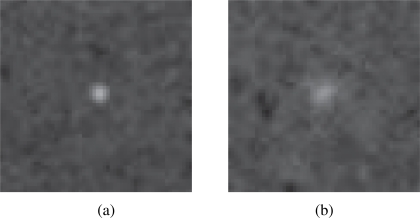
Fluorescent particle in (a) clear and (b) turbid water.

Figure 4 shows two representative images from the experimental campaign displaying the particle in clear and turbid water conditions. More specifically, Figure 4(a) shows the fluorescent bead in a clear water suspension at an immersion depth of 4 mm. It can be noted that the particle pixel area is approximately equal to the reference area. On the other hand, Figure 4(b) displays the particle in a 1 g/L clay-water suspension at a depth of 6 mm. The effects of clay and depth result into spreading of the bead boundaries in the image. This is mainly due to clay particles in the proximity of the bead scattering the fluorescence emissions. As a result, fluorescent spheres appear larger in the second image. On the other hand, fluorescent particles suspended in low clay concentrations and shallow depths appear smaller and with sharper boundaries as compared to turbid water conditions. In the former case, images show a meagre number of clear pixels with respect to the background frames.

**Figure 5.**
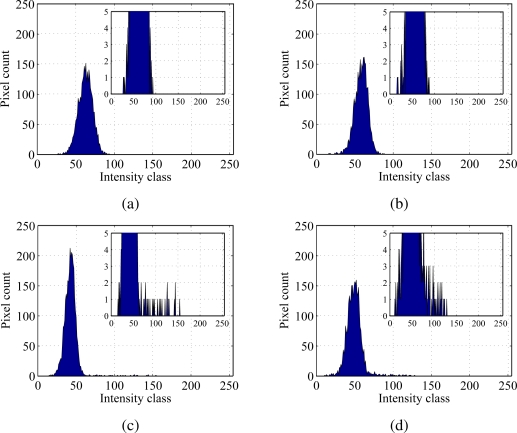
Intensity histograms relative to (a) clear water and (b) turbid water condition backgrounds. Histograms relative to particle in (c) clear water and (d) turbid water condition. Clay concentration is set to 0 g/L in (a) and (c) and to 1 g/L in (b) and (d). Water depth is 4 mm in (a) and (c) and 6 mm in (b) and (d).

The analysis of the particle visibility in turbid water is qualitatively conducted by utilizing histograms obtained from 8-bit gray-scale frames. Accordingly, 256 classes of intensity are considered. The 8-bit gray-scale image is extracted from the original RGB (red, green, blue) picture by retaining only the green channel [51]. Figure 5 reports histograms pertaining to particle frames and corresponding backgrounds in Figure 4. Note that histogram bins related to low intensity classes refer to darker colors in the images. Bins corresponding to higher intensity classes may instead be ascribed to the presence of fluorescent particles.

Figure 5(a,c) represent the background and particle frame histograms, respectively, for clear water conditions. Figure 5(b,d) display the background and particle frame histograms for the 1 g/L suspension. The insets magnify low pixel count bins. By comparing these insets, it can be noted that Figures 5(c,d) present non-zero pixel counts at higher intensity classes. This effect is due to the presence of the fluorescent particle and therefore represents the fluorescent emissions. In particular, the inset in Figure 5(d), corresponding to the turbid concentration, displays non-zero pixel counts at intensity classes around 100–120. Conversely, the inset for clear water in Figure 5(c) depicts non-zero counts up to the neighborhood of class 150. This can be attributed to bead boundaries spreading in turbid water. On the other hand, background histograms in Figure 5(a,b) show peaks that are slightly shifted towards brighter classes when compared to their counterparts in Figure 5(c,d). This phenomenon is likely due to light scattering through clay particles. In addition, the peak in Figure 5(c) is higher than the corresponding one in the background frame, as shown in Figure 5(d).

Information from histograms can be lumped into appropriate global parameters to quantify the particle visibility against the background, including the effective particle boundary spreading and brightness. More specifically, the following index 𝒢 is adopted to synthetically describe the effect of fluorescence on the overall frame tonal distribution:

(2) $$𝒢=\frac{\sum_{i\in 𝒥}c_{i}n_{i}}{\sum_{i\in 𝒥}n_{i}},\;\;\;\;\;𝒥=\{i\in \{0,\;1,\;\ldots ,\;255\}:\;n_{i}>0\}$$

with

(3) $$n_{i}=n_{i}^{p}-n_{i}^{b}.$$

Here, *c_i_* represents the intensity classes from 0 to 255 and *n_i_* the pixel count on each class of the histogram obtained by subtracting the background, that is, $n_{i}^{b}$ from the particle frame, that is, $n_{i}^{p}$, and including in the summation only the classes with positive pixel counts. Tones corresponding to the particle are expected to be in the higher intensity classes, while background pixels pertain to darker classes. By subtracting $n_{i}^{b}$ from $n_{i}^{p}$, positive and negative pixel counts are expected to occur for brighter and darker classes, respectively. Therefore, by discarding negative pixel counts, the particle average intensity tone is identified. For instance, by subtracting the background, see Figures 5(a,b), to the particle frame, Figures 5(c,d), the particle is automatically isolated from the background and its contribution to the frame tonal distribution is quantitatively estimated. It should be noted that analogous background subtraction techniques find also application in the detection of boundaries of moving shapes, as discussed for example in [52,53]. The index 𝒢 is expected to increase for high clay concentrations, where the bead boundaries tend to spread, thus resulting in larger pixel counts at brighter tones. In addition, note that the index 𝒢 is highly dependent on the background illumination conditions and color homogeneity.

**Figure 6.**
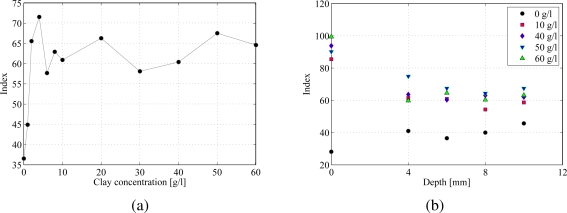
Index variation as a function of (a) clay concentration at an immersion depth of 6 mm and (b) bead immersion depth.

In Figure 6(a), the index 𝒢 is evaluated for increasing clay-water concentrations at the bead immersion depth of 6 mm. The parameter is minimized for clear water since the particle is very defined and its area comprises only a few pixels. On the other hand, as the turbidity increases, the number of brighter pixels increases and the index varies accordingly. The behavior shown in Figure 6(a) displays qualitative similarities with the experimental evidence collected from the fluorescence measurements in Section 3.1. In particular, when comparing Figures 3(b) and 6(a), it can be noted that the output voltage and the index 𝒢 present a peak at the same concentration of 4 g/L. At very large concentrations, the index tends to increase due to the effect of the bead boundaries spreading, whereas the output voltage decreases as the photoresistors excitation level decreases.

In Figure 6(b), 𝒢 is estimated for selected concentrations at varying bead immersion depths. It is here noted that different concentrations display a comparable behavior in the bead boundary spreading. As the turbid layer becomes thicker, the number of brighter pixels tends to increase. In addition, the effect of immersion depth on the visibility is similar to the increase in clay concentration.

Index values at the depth of 0 mm are also reported in Figure 6(b). In this case, data are obtained by positioning both the UV lamp and the camera at the relatively large distance of 40 cm above the sample. This selection simulates a practical implementation of the detection system for in-situ applications. Note that index values corresponding to depths of 0 mm tend to cluster around high intensity classes. However, the value of 𝒢 that refers to clear water is much smaller than numerical values found in turbid water scenarios, due to effects of reflections in water. Indeed, in clear water, reflections produce a relatively large number of pixels which are as bright as the particle pixels and contribute to the reduction of 𝒢.

## 4. Discussion

In this study, particle detection is performed in controlled laboratory conditions, such as placid water and dark ambient illumination, to minimize noise from water turbulence and spurious ambient light sources and, consequently, to isolate the fluorescence emissions. Nonetheless, in-situ applications of this technology can be influenced by typical natural environment factors such as ambient light and flow velocity. In this Section, some of the most significant among these effects are discussed and experimentally investigated.

The effect of ambient light can highly alter the images and therefore compromise the particle detection process. More specifically, isolating fluorescent emissions in the frames under the high intensity of sunlight may be much more complex than in dark conditions. In addition, the green component of sunlight spectrum is not blocked by the filter and contributes to increase the actual intensity values. This phenomenon may result in saturation of the higher tones of the image and, therefore, in inherent difficulties in distinguishing among particles’ luminescence and background brightness by mere histogram inspection.

**Figure 7.**
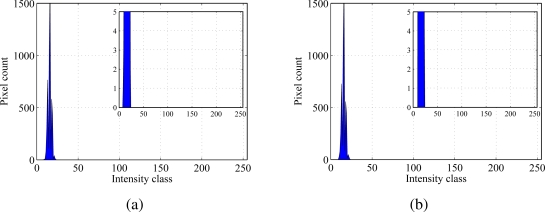
(a) Particle frame histogram and (b) background frame histogram. Pictures are recorded at a shutter speed of 1/30, 000 s.

Experiments are performed in daylight conditions to investigate how external light can affect the proposed technology. Figure 7 presents particle and background frame histograms for a concentration of 2 g/L and at an immersion depth of 4 mm. An 811 × 508 pixel Hitachi KP-D20 B CCD camera is used for the experiments. The camera is located at approximately 30 cm below the particle; the UV lamp is positioned at 10 cm above the sample. A 540 nm filter is used to isolate the fluorescent emissions. Images are acquired with shutter speed kept at the minimum available value of 1/30, 000 s. This results in excessively dark images and poor histograms that do not allow for particle identification.

Better pictures can be obtained by adjusting the acquisition parameters and, therefore, identifying the light emitted by the particles. To address this issue, a Labview VI is developed to allow for camera self-calibration in response to environment conditions. More specifically, during the acquisition, camera shutter speed is iteratively varied starting from the minimum to the optimal speed that allows the particle to be detected. This adaptation is implemented by performing a so-called particle analysis procedure on the acquired frames. Initially, a threshold is applied to the image to isolate bright from dark pixels. After pixels belonging to the specified intensity threshold are identified, they are connected into so-called objects according to their adjacency in the image and their area is computed. Finally, the particle analysis procedure yields the number of objects matching the user-calibrated fluorescent bead’s dimensions [54]. Optimal exposure time is found as the bead is recognized within the image. Images used for particle detection are stored for possible further processing and data extraction.

**Figure 8.**
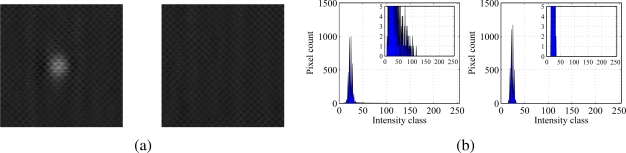
(a) Particle (left) and background (right) in ambient light and (b) corresponding histograms. Pictures are recorded at a shutter speed of 1/100 s.

Figure 8 depicts the same conditions shown in Figure 7 recorded after camera self-calibration at a shutter speed of 1/100 s. It can be noted that the bead is visible just by inspection and histograms allow for a good estimation of the index 𝒢.

Another major challenge in the detection of hydrologic tracers is represented by stream flows. At high flow rates, sensing and tracking small objects can be extremely complex [13]. In addition, further difficulties arise from the effect of Stokes drag forces that may result in relevant immersion depths [44] and from the reflection noise due to the presence of waves and turbulence [55].

**Figure 9.**
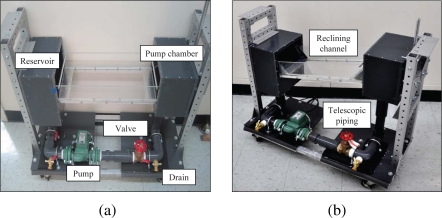
(a) Miniature channel front and (b) side view displaying the reclining channel. Telescopic piping allows the pump chamber to be raised.

As a proof of concept of the applicability of the proposed detection technology in critical field measurements, additional experiments are performed in dynamic conditions. Within this feasibility study, a miniature water channel is used to evaluate the detectability of the particles in flowing water, see Figure 9(a,b). In this channel, water flows through an acrylic 50 cm × 20 cm × 10 cm channel. The structure is placed on an aluminum scaffolding and attached to a reservoir and pump chamber that can slide on a guide rail to freely move up and down. The whole system is sealed with Polyvinyl Chloride (PVC) sheets and cement. Telescopic piping connects the pump to the pump chamber to allow for different inclinations of the channel. Piping and pump are offset from the bottom of the channel to allow experimental equipment to be placed below the channel. A 1/25 hp compact cast iron centrifugal pump circulates water into the system. Light foam and wood panels are anchored to the pump support to prevent excessive vibrations. Water flow is regulated through a valve gate attached to the piping system which restricts the efflux cross-section.

**Figure 10.**
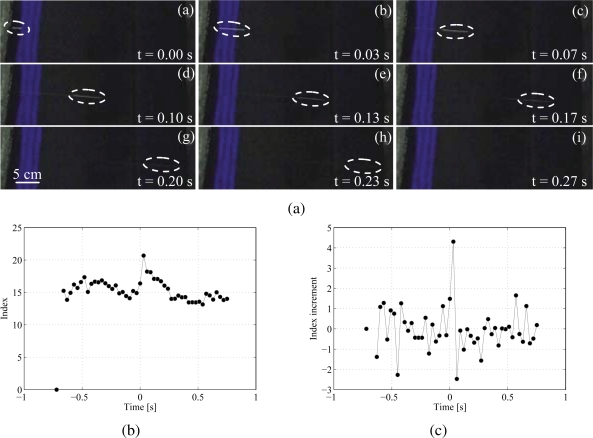
(a) Snapshots of particle motion, (b) index variation, and (c) index incremental changes during bead motion. In (a), the tracked particle position is highlighted inside the dashed ellipse.

In the experiments, a few fluorescent particles are deployed into the channel and illuminated by the UV light placed at a distance of approximately 10 cm from the water surface. A low-cost CMOS Canon VIXIA HG20 camcorder is used for video acquisition. The camcorder resolution is 1, 920×1, 080 pixels and its acquisition frequency is fixed at 30 Hz. The camera is located at 50 cm from the water and frames are recorded in dark surrounding conditions, as displayed in Figure 10(a).

A quantitative identification of the particles transit in the channel is performed by evaluating the index 𝒢 on a representative sequence of frames. The first picture of the recorded sequence is arbitrarily selected as reference background frame, see the first marker in Figures 10(b,c), and the index 𝒢 is computed on each of the subsequent images. Figure 10(b) displays the calculated values against time, where the origin of time is set in correspondence of the first frame in which the bead is observed, consistently with Figure 10(a). The non-zero mean value of approximately 15 in Figure 10(b) corresponds to the average intensity class of the frame tone distribution. The bead transit in the channel can therefore be associated with a sudden increase in the value of the index at time equal to zero. Before and after the bead transit, values of 𝒢 are significantly lower and approximately constant. Figure 10(c) presents the index incremental change between two consecutive frames for the same time interval. A clear spike from smaller zero-mean oscillations occurs in the index variation when the camcorder senses the fluorescence emission.

As a concluding remark, transit speed ν estimated through image analysis leads to a value of approximately 1 m/s, as the particle flows in 0.3 s through a channel length of approximately 30 cm. Observed values are consistent with the predictions obtained by using the classical Manning’s equation for open-channel flow [56], that is,

(4) $$v=R_{h}^{2/3}S_{0}^{1/2}/n$$

where *R_h_* and *S*_0_ denote the channel hydraulic radius and slope, respectively. For the experimental conditions depicted in Figure 9, *R_h_* is approximately equal to 0:009 m and *S*_0_ to 0:17. Artificially lined channels present Manning’s resistance coefficients *n* in the range of 0.010–0.025 s/m^1/3^. For such values, bounds for the transit velocity can be estimated as 0.71–1.78 m/s.

## 5. Conclusions

In this paper, the feasibility of off-the-shelf buoyant fluorescent particles for tracing high turbidity surface water flows is assessed. Particle fluorescence is quantitatively measured for different levels of turbidity by using an array of photoresistors. Experiments show that particle fluorescence is well detected for clay concentrations as high as 60 g/L. In addition, particle detection and localization in turbid water flows is performed by using image processing tools. In particular, spreading of the bead average fluorescence intensity for increasing turbidity is described through a suitably defined global index. Results obtained with the two independent methodologies exhibit comparable trends and qualitative similarities. The overall experimental study suggests that particles are detectable in critically turbid water flows. The potential of the proposed detection technology for application in natural environments is also discussed along with possible technological ameliorations for improving bead visibility in daylight and turbulent flows. Despite the unfavorable conditions, the proposed technology demonstrates high reliability and is expected to significantly contribute to the enhancement of the established practice in surface water flow measurements and, in broader terms, to sensing technologies for environmental studies.

Future experimental work will be devoted to the fabrication of low-cost in-house developed tracer particles from different environmentally-friendly materials and alternative fluorescent dyes. In addition, optimal tracer development will be informed by analytical investigation of the motion of particles in multiphase flows, towards formulation of tracer design criteria for accurate monitoring of highly turbulent flows.

## References

[1] Chow VT, Maidment DR, Mays LW (1988). Applied Hydrology.

[2] Bras RL (1989). Hydrology: An Introduction to Hydrologic Science.

[3] Bansal MK (1971). Dispersion in natural streams. ASCE J Hydraul Div.

[4] Piccarreta M, Faulkner H, Bentivenga M, Capolongo D (2006). The influence of physico-chemical material properties on erosion processes in the badlands of Basilicata, Southern Italy. Geomorphology.

[5] Wainwright J (1996). Infiltration, runoff and erosion characteristics of agricultural land in extreme storm events, SE France. Catena.

[6] Kondolf MG, Piégay H (2003). Tools in Fluvial Geomorphology.

[7] Rulli MC, Rosso R (2005). Modeling catchment erosion after wildfires in the San Gabriel Mountains of southern California. Geophys Res Lett.

[8] Chirico GB, Medina H, Romano N (2007). Uncertainty in predicting soil hydraulic properties at the hillslope scale with indirect methods. J Hydr.

[9] Gasparini NM, Tucker GE, Bras RL (2004). Network-scale dynamics of grain-size sorting: Implications for downstream fining, stream-profile concavity, and drainage basin morphology. Earth Surf Process Landf.

[10] Istanbulluoglu E, Yetemen O, Vivoni ER, Gutierrez-Jurado HA, Bras RL (2008). Eco-geomorphic implications of hillslope aspect: Inferences from analysis of landscape morphology in central New Mexico. Geophys Res Lett.

[11] Tucker GE, Bras RL (1998). Hillslope processes, drainage density, and landscape morphology. Water Resour Res.

[12] Leibundgut C, Maloszewski P, Külls C (2009). Tracers in Hydrology.

[13] Jodeau M, Hauet A, Paquier A, Le Coz J, Dramais G (2008). Application and evaluation of LS-PIV technique for the monitoring of river surface velocities in high flow conditions. Flow Meas Instrum.

[14] Kim Y, Muste MVI, Hauet A, Krajewski WF, Kruger A, Bradley AA (2008). Stream discharge using mobile large-scale particle image velocimetry: A proof of concept. Water Resour Res.

[15] Calkins D, Dunne T (1970). A salt tracing method for measuring channel velocities in small mountain streams. J Hydr.

[16] Creutin JD, Muste M, Bradley AA, Kim SC, Kruger A (2003). River gauging using PIV techniques: A proof of concept experiment on the Iowa River. J Hydr.

[17] Lyon SW, Desilets SLE, Troch PA (2008). Characterizing the response of a catchment to an extreme rainfall event using hydrometric and isotopic data. Water Resour Res.

[18] Pilgrim DH (1966). Radioactive tracing of storm runoff on a small catchment: I. Experimental technique. J Hydr.

[19] Planchon O, Silvera N, Gimenez R, Favis-Mortlock D, Wainwright J, Le Bissonnais Y, Govers G (2005). An automated salt-tracing gauge for flow-velocity measurement. Earth Surf Process Landf.

[20] Botter G, Milan E, Bertuzzo E, Zanardo S, Marani M, Rinaldo A (2009). Inferences from catchment-scale tracer circulation experiments. J Hydr.

[21] Holecek J, Vocel J

[22] Hubbard EF (1982). Measurement of Time of Travel and Dispersion in Streams by Dye Tracing.

[23] Hassan MA, Ergenzinger P (2003). Tools in Fluvial Geomorphology.

[24] Pilgrim DH (1975). Travel times and nonlinearity of flood runoff from tracer measurements on a small watershed. Water Resour Res.

[25] Wienhöfer J, Germer K, Lindenmaier F, Färber A, Zehe E (2009). Applied tracers for the observation of subsurface stormflow at the hillslope scale. Hydrol Earth Syst Sci.

[26] Waldon MG (2004). Estimation of average stream velocity. J Hydraul Eng.

[27] Johnson D, Pattiaratchi C (2004). Application, modelling, and validation of surfzone drifters. Coast Eng.

[28] Stockdale RJ, McLelland SJ, Middleton R, Coulthard TJ (2008). Measuring river velocities using GPS River Flow Tracers (GRiFTers). Earth Surf Process Landf.

[29] Raffel M, Willert CE, Wereley ST, Kompenhans J (2007). Particle Image Velocimetry A Practical Guide.

[30] Bradley AA, Kruger A, Meselhe EA, Muste MVI (2002). Flow measurement in streams using video imagery. Water Resour Res.

[31] Adrian RJ (2005). Twenty years of particle image velocimetry. Exp Fluid.

[32] Angarita-Jaimes D, Ormsby M, Chennaoui M, Angarita-Jaimes N, Towers C, Jones A, Towers D (2008). Optically efficient fluorescent tracers for multi-constituent PIV. Exp Fluid.

[33] Meselhe EA, Peeva T, Muste MVI (2004). Large scale particle image velocimetry for low velocity and shallow water flows. J Hydrol Eng.

[34] Jin S, Huang P, Park J, Yoo JY, Breuer KS (2004). Near-surface velocimetry using evanescent wave illumination. Exp Fluid.

[35] Sarraf CE (2000). Diagnostic and Therapeutic Antibodies.

[36] Hoffmann RM (2008). Imaging in mice with fluorescent proteins: From macro to subcellular. Sensors.

[37] Orcutt KM, Ren S, Gundersen K (2009). Detecting proteins in highly autofluorescent cells using quantum dot antibody conjugates. Sensors.

[38] Tiwari DK, Tanaka SI, Inouye Y, Yoshizawa K, Watanabe TM, Jin T (2009). Synthesis and characterization of anti-HER2 antibody conjugated CdSe/CdZnS quantum dots for fluorescence imaging of breast cancer cells. Sensors.

[39] Milstein AB, Kevin SO, Webb J, Bouman CA, Zhang Q, Boas DA, Millane RP (2003). Fluorescence optical diffusion tomography. Appl Optics.

[40] Pedocchi F, Martin J, García MH (2008). Inexpensive fluorescent particles for large-scale experiments using particle image velocimetry. Exp Fluid.

[41] Weitsman YJ, Elahi M (2000). Effects of fluids on the deformation, strength and durability of polymeric composites—An overview. Mech Time-Depend Mater.

[42] Lenzi MA, Marchi L (2000). Suspended sediment load during floods in a small stream of the Dolomites (Northeastern Italy). Catena.

[43] Lambe TW, Whitman RV (1969). Soil Mechanics.

[44] Crowe C, Sommerfield M, Tsuji Y (1998). Multhiphase Flows with Droplets and Particles.

[45] Merzkirch W (1974). Flow Visualization.

[46] Defina A, Petruzzo P (2010). Floating particle trapping and diffusion in vegetated open channel flow. Water Resour Res.

[47] Merritt DM, Wohl EE (2002). Processes governing hydrochory along rivers: Hydraulics, hydrology, and dispersal phenology. Ecol Appl.

[48] Groves JH, Williams DG, Caley P, Norris RH, Caitcheon G (2009). Modelling of floating seed dispersal in a fluvial environment. River Res Appl.

[49] Cospheric LLC http://www.cospheric-microspheres.com/default.asp/.

[50] Alciatore DG, Histand B (2003). Introduction To Mechatronics And Measurement Systems.

[51] Gonzalez RC, Woods RE, Eddins SL (2004). Digital Image Processing using MATLAB.

[52] Hou L, Kagami S, Hashimoto K (2010). Illumination-based synchronization of high-speed vision sensors. Sensors.

[53] Varcheie PDZ, Sills-Lavoie M, Bilodeau GA (2010). A multiscale region-based motion detection and background subtraction algorithm. Sensors.

[54] (1999). National Instruments. IMAQ Image Processing Manual.

[55] Weitbrecht V, Kühn G, Jirka GH (2002). Large scale PIV-measurements at the surface of shallow water flows. Flow Meas Instrum.

[56] Munson BR, Young DF, Okiishi TH (2002). Fundamental Fluid Mechanics.

